# Optimal cut‐off scores for mild cognitive impairment and dementia of the Spanish version of the Montreal Cognitive Assessment in Costa Rican older adults

**DOI:** 10.1002/alz.083981

**Published:** 2025-01-03

**Authors:** Carolina Boza, Jose Pablo Ulate‐Aguilar, Shirley Rojas‐Salazar, Norbel Román, Arjun V. Masurkar

**Affiliations:** ^1^ Universidad de Costa Rica, San José Costa Rica; ^2^ CIHATA, University of Costa Rica, San José Costa Rica; ^3^ School of Statistics, University of Costa Rica, San José Costa Rica; ^4^ Department of Neurology, San Juan de Dios Hospital, San José Costa Rica; ^5^ Alzheimer’s Disease Research Center, New York University Langone Health, New York, NY USA

## Abstract

**Background:**

Alzheimer’s disease and related dementias (AD/ADRD) burden in Costa Rica is expected to become one of the highest in the region. Early detection will help optimize resources and improve primary care interventions. The Montreal Cognitive Assessment (MoCA) has shown good sensitivity for detection of mild cognitive impairment (MCI), but specificity varies depending on the population. This motivates the analysis of different cut‐offs to minimize false‐positive classifications in a Costa Rican sample for its use in clinical settings.

**Methods:**

Data was analyzed from 516 memory clinic outpatients (148 cognitively normal, 260 MCI, 108 mild dementia; mean age 66.3 ± 10.8 years) who underwent complete neurological and neuropsychological assessment and were diagnosed by consensus. Optimal MoCA cut‐off scores were identified using the Youden’s Index, balanced cut‐offs, and specificity scores.

**Results:**

Overall, a cut‐off score of ≥23 showed better accuracy to distinguish between NC and MCI (sensitivity 73%, specificity 83%). When analyzed by educational levels, cut‐off scores of ≥21 showed better accuracy for ≤6 years (sensitivity 80%, specificity 76%), ≥23 for 7‐12 years (sensitivity 86%, specificity 76%) and ≥24 for ≥12 years (sensitivity 70%, specificity 85%). For distinguishing MCI from mild dementia, the optimal overall cut‐off score was ≥15 (sensitivity 65%, specificity 85%). When stratified by years of education, cut‐off scores of ≥14 showed better accuracy for ≤6 years (sensitivity 70%, specificity 88%), ≥15 for 7‐12 years (sensitivity 79%, specificity 95%) and ≥17 for ≥12 years (sensitivity 67%, specificity 93%).

**Conclusions:**

A MoCA cut‐off score of ≥23 in Costa Rican population showed better diagnostic accuracy and may reduce the false positive rate. Our findings may be helpful for primary care clinical settings and further referral criteria.

**Keywords:**

MoCA, Montreal Cognitive Assessment

